# Acidic cellular microenvironment modifies carcinogen-induced DNA damage and repair

**DOI:** 10.1007/s00204-016-1907-4

**Published:** 2016-12-22

**Authors:** Q. Shi, L. Maas, C. Veith, F. J. Van Schooten, R. W. Godschalk

**Affiliations:** Department of Pharmacology and Toxicology, NUTRIM School for Nutrition and Translational Research in Metabolism, Maastricht University, PO Box 616, 6200 MD Maastricht, The Netherlands

**Keywords:** Acidic pH, Benzo[a]pyrene, Cytochrome P450 (CYP1A1), DNA damage, NER

## Abstract

Chronic inflammation creates an acidic microenvironment, which plays an important role in cancer development. To investigate how low pH changes the cellular response to the carcinogen benzo[a]pyrene (B[a]P), we incubated human pulmonary epithelial cells (A549 and BEAS-2B) with nontoxic doses of B[a]P using culturing media of various pH’s (extracellular pH (pH_e_) of 7.8, 7.0, 6.5, 6.0 and 5.5) for 6, 24 and 48 h. In most incubations (pH_e_ 7.0–6.5), the pH in the medium returned to the physiological pH 7.8 after 48 h, but at the lowest pH (pH_e_ < 6.0), this recovery was incomplete. Similar changes were observed for the intracellular pH (pH_i_). We observed that acidic conditions delayed B[a]P metabolism and at *t* = 48 h, and the concentration of unmetabolized extracellular B[a]P and B[a]P-7,8-diol was significantly higher in acidic samples than under normal physiological conditions (pH_e_ 7.8) for both cell lines. Cytochrome P450 (*CYP1A1/CYP1B1*) expression and its activity (ethoxyresorufin-O-deethylase activity) were repressed at low pH_e_ after 6 and 24 h, but were significantly higher at *t* = 48 h. In addition, a DNA repair assay showed that the incision activity was ~80% inhibited for 6 h at low pH_e_ and concomitant exposure to B[a]P. However, at *t* = 48 h, the incision activity recovered to more than 100% of the initial activity observed at neutral pH_e_. After 48 h, higher B[a]P-DNA adduct levels and γ-H2AX foci were observed at low pH samples than at pH_e_ 7.8. In conclusion, acidic pH delayed the metabolism of B[a]P and inhibited DNA repair, ultimately leading to increased B[a]P-induced DNA damage.

**Electronic supplementary material:**

The online version of this article (doi:10.1007/s00204-016-1907-4) contains supplementary material, which is available to authorized users.

## Introduction

Acidic microenvironments have been found in tumors and in chronically inflamed tissues (Kato et al. 2013; Lardner 2001). During tumorigenesis, the main cause for the acidic microenvironment is anaerobic glycolysis, which produces acidic metabolites (e.g., lactic acid) (Kato et al. 2013). Under chronic inflammatory conditions, the local extracellular pH can decrease to pH 5.5 or even lower, through release of acidic compounds by inflammatory cells, including hypochloric acid (HOCl) and by subsequent damaging of surrounding tissues. (Ganong 1985; Mollon et al. 2008). Endogenous airway acidification is related to inflammation in for instance chronic obstructive pulmonary disease (COPD) patients, whose exhaled breath condensate pH is related to disease severity (Papaioannou et al. 2011). Many cancers are preceded and accompanied by inflammatory surroundings explaining partly the association between COPD and lung cancer (Bozinovski et al. 2015; Knaapen et al. 2006). Inflammation may thus be a factor involved in initiation as well as progression of tumorigenesis (Hanahan and Weinberg 2011). Although the mechanism behind the development of both chronic inflammation and cancer is different, a common characteristic is an acidic microenvironment. At present, there are few studies and thus little knowledge about the underlying mechanism of how a low pH can modulate DNA damage formation and subsequent tumorigenesis. Nonetheless, there are some studies that suggest that acidic pH can also change the response of cells to exogenous genotoxins, such as polycyclic aromatic hydrocarbons (PAH). For instance, acidic conditions promoted carcinogenesis in the hamster cheek pouch after exposure to the tumor initiator 9,10-dimethyl-1,2-benzanthracene (DMBA) (Adams et al. 2000). In addition, a combination of hypoxia and low pH diminished DNA repair and increased mutagenesis in mammalian cells (Yuan et al. 2000). Interestingly, a dose-dependent inhibition of nucleotide excision repair (NER) was also reported after exposure of cells to HOCl, which is expected to lower the extracellular pH (Gungor et al. 2007).

**Fig. 1:**
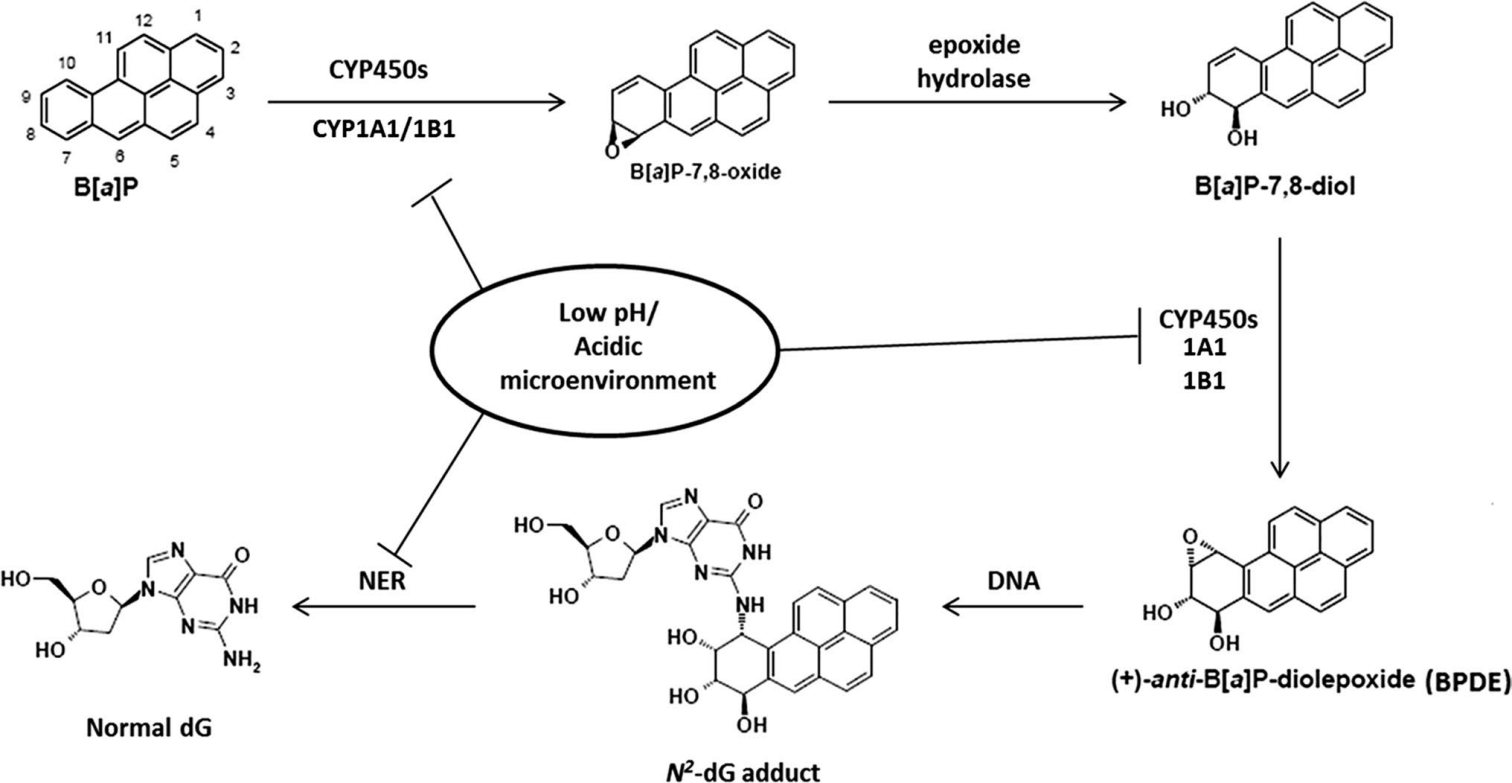
General B[a]P metabolism pathway and the role of low pH/acidic microenvironment. This figure is adapted from Shi et al. (2015) with some modifications

Inhibition of NER capacity would result in accumulation of DNA damage after exposure to DNA damaging compounds such as PAH. One environmentally abundant PAH is benzo[a]pyrene (B[a]P), and human exposure to B[a]P is inevitable, since B[a]P is present in cigarette smoke, diesel exhaust, furnace burning of coal or oil and in food (Godschalk et al. 2002; Kazerouni et al. 2001; Tancell et al. 1995). When B[a]P enters the cell, it binds to the aryl hydrocarbon receptor (AhR) and this leads to the up-regulation of cytochrome P450 isoforms CYP1A1 (Spink et al. 2002). By inducing the expression of *CYP1A1*, B[a]P induces its own metabolism (Baird et al. 2005). Eventually, B[a]P may be activated to the ultimate carcinogen B[a]P-diol epoxide (BPDE) which can covalently bind to DNA to form pro-mutagenic DNA adducts (Gelboin 1980) (Fig. 1). Interestingly, the majority of in vitro studies on B[a]P-induced genotoxicity were conducted at neutral physiological pH (7.4–7.8). However, human exposure to B[a]P via cigarette smoke and air pollution is often characterized by inducing inflammation. Therefore, the combined effects of carcinogenic exposures with inflammation are worth studying.

An acidic pH changes multiple characteristics of cells, and the influence on the molecular events induced by B[a]P exposure is still unclear. In the current study, we focus on lung cells because the lung is probably one of the most vulnerable organs for exposure to both inflammation and B[a]P. Therefore, we incubated human adenocarcinoma cells (A549) and human bronchial epithelial cells (BEAS-2B) under different extracellular pH conditions with B[a]P, and we investigated whether acidic pH can alter metabolic enzyme activity, B[a]P metabolite formation, DNA repair and ultimately B[a]P-induced DNA damage.

## Materials and methods

### Cell culture and treatment

A549 cells (human epithelial lung carcinoma cells) and BEAS-2B cells (human bronchial epithelium cells) were purchased from the American Tissues Culture Collection (ATCC, Rockville, MD, USA) and were authenticated by ATCC using short tandem repeat profiling (www.atcc.org/STR%20Database.aspx). A549 cells were cultured in RPMI 1640 Medium (Sigma-Aldrich) supplemented with 5% heat inactivated fetal calf serum (FCS, Gibco Invitrogen, Breda, The Netherlands) and 1% penicillin/streptomycin (Sigma) in T75 flasks. BEAS-2B cells were cultured in Dulbecco’s Modified Eagle Medium/F-12 Nutrient Mixture (Ham) (DEME/F-12, Gibco, Carlsbad, CA, USA) supplemented with 10% heat inactivated fetal bovine serum (FBS, Gibco) and 1% penicillin/streptomycin (Sigma) in T75 flasks. All cells were cultured under a humidified atmosphere containing 5% CO_2_ at 37 °C, and passages between 15 and 30 were used. Cells were routinely screened for mycoplasma contamination by a Mycoplasma Detection Kit (InvivoGen, The Netherlands). Different pH in media (pH 5.5, 6, 6.5, 7 and 7.8) was achieved by adding 1 N HCl to the medium. All chemicals were purchased from Sigma-Aldrich unless stated otherwise. All cells were cultured with different pH media and 1 μM B[a]P, which was dissolved in DMSO (final concentration did not exceed 0.5%). After 6-, 24- and 48-h treatment, the medium were harvested and cells were collected as pellets, and all materials were stored at −20 °C until further analysis.

### Measurement of cell cytotoxicity and viability

Cytotoxicity of different pH media and B[a]P was evaluated by using the 3-(4,5-dimethylthiazol-2-yl)-2,5-diphenyl-tetrazolium bromide (MTT) colorimetric assay described by Mosmann (Mosmann 1983). Briefly, cells were plated into 96-well plates (Costar, Cambridge, MA) at 1 × 10^4^ cells/100 μl medium and cultured for 2 days. At confluence, cells were exposed to different pH media (pH 5.5, 6, 6.5, 7 and 7.8) and 1 μM B[a]P for 6, 24 and 48 h. After incubation, all cells were washed with 100 μl 37 °C Hank’s balanced salts (HBSS) and MTT (0.5 mg/ml), which was dissolved in phosphate-buffered saline (PBS), was added. Cells were incubated in the dark at 37 °C for 1 h. Then, solutions were removed, and formazan crystals were dissolved in 200 μl DMSO for 30 min at room temperature. Finally, absorption was measured by using a microplate reader at 540 nm (BioRad, Veenendaal, The Netherlands), and the cell viability was expressed as percentage of control (i.e., ‘physiological’ pH 7.8).

Since acidic pH might influence the results of the MTT assay (Plumb et al. 1989), we additionally performed a trypan blue exclusion test as an additional cell viability indicator. Briefly, cells were cultured in 24-well plates (Costar, Cambridge, MA) at 1 × 10^5^ cells/500 μl medium and cultured for 2 days. As described above, cells were treated with different pH media and 1 μM B[a]P for 6, 24 and 48 h when the cells in each well reached to 90% confluence. After harvesting the cells, cells were resuspended in 1 ml warm HBSS. A total volume of 1 ml contained 0.5 ml of 0.4% trypan blue solution with additional 0.3 ml of HBSS and 0.2 ml of the cell suspension. The cell viability was presented as percentage of the ratio of total cells (stained and unstained) divided by total viable cells (unstained) × 100.

### Cell proliferation

Cells were cultured in 6-well plates (Costar, Cambridge, MA) at 1.2 × 10^6^ cells/3 ml in different pH media (pH 5.5, 6, 6.5, 7 and 7.8) for 2 days. After trypsinization, cells were collected and resuspended in 1 ml warm HBSS. Ten μl of cell suspension was transferred to a hemacytometer to microscopically count the cells. Each well was counted twice.

### Extracellular and Intracellular pH measurement

Extracellular pH (pHe) in media was measured with a pH meter (HI 2210, HANNA, Australia). The medium (with and without cells) at each time point was collected, and the pH was measured immediately in an aliquot of 4 ml. For intracellular pH (pH_i_) measurement, 1 × 10^4^ cells/200 μl medium was incubated in 96-well plates. When the cells reached 70–80% confluency, the attached cells were loaded with the pH_i_-sensitive fluorescent dye 2′,7′-bis(2-carboxyethyl)-5,6-carboxyfluoresceinacetoxymethyl ester (BCECF-AM) by incubation with 5 μM of the cell-permeant dye BCECF-AM for 30 min. The loaded cells were washed two times to minimize extracellular BCECF. After that, cells were cultured with different pH media (pH 7.8, 7.0, 6.5, 6.0 and 5.5) for 6, 24 and 48 h. Fluorescence was measured using a spectrofluorometer (Spectra max m2, MDS, CA) at 37 °C and with excitation wavelength pairs of 505/439 nm and an emission wavelength of 535 nm for 10 min. The ratio of fluorescence of BCECF at 505–439 nm is considered as the function of pH_i_ (Whiteman et al. 2002).

The pH calibration curve was obtained as described by Lo et al. (1995), by incubation of BCECF-loaded cells with additional depolarizing buffer containing the proton ionophore nigericin (10 μM) for 10 min; under this condition, the pH_i_ is equal to the pH of the medium. The pH medium was made from 50 mM Tris–HCl buffer and titrated from 7.8 to 5.5 by additions of 1 M HCl, respectively. The final added volume of acid solution is less than 2% of total volume, and this amount was unlikely to create an osmotic effect. The pH calibration curve was constructed from four independent sets of data. The ratio of the fluorescence at the dual excitation wavelengths (505 and 439 nm) was converted into pH_i_.

### HPLC fluorescence analysis of B[a]P metabolites

B[a]P and its metabolites were extracted and identified as described by Schults et al. (2013b). Briefly, 5 ml of cell medium was mixed by 1 ml of ethyl acetate for 20 min, followed by centrifugation (10 min, 980 g). The top layer was transferred to a new tube and evaporated under nitrogen. Finally, the evaporated residue was redissolved in 0.5 ml methanol and analyzed by HPLC-FD using a Gynkotek P580A HPLC system (Separations Analytical Instruments, Hendrik-Ido-Ambacht, The Netherlands) consisting of a Spark SP830 autosampler (Spark Holland, Emmen, The Netherlands) with a PerkinElmer LS-30 programmable fluorescence detector (Perkin Elmer, Foster City, CA, USA) operated at excitation/emission wavelengths 257/350 nm. A standard mix that contained 50 ng/ml of B[a]P-9,10-diol, B[a]P-7,8-diol and 3-OH-B[a]P (Midwest Research Institute, Kansas City, MO, USA) were injected for quantitation purposes.

### Real-time quantitative PCR

For investigating *CYP1A1* and *CYP1B1* gene expression, cell pellets were collected after 6-, 24- and 48-h incubation. Total RNA was isolated and purified by using the RNeasy^®^ Mini Kit (Qiagen Westburg, Leusden, The Netherlands) in combination with DNase treatment (Qiagen). cDNA was prepared by using the iScript™ cDNA Synthesis kit (BioRad, CA, USA), starting with 500 ng of RNA. cDNA was 10 × diluted in water before use in the RT-PCR. The reaction was conducted using a BioRad MyiQ iCycler Single Color RT-PCR detection system using iQ™ SYBR^®^ Green Supermix (BioRad), 5 μl diluted cDNA and 0.3 μM *β*-*actin*, *GAPDH*, *CYP1A1* or *CYP1B1* primers (for specific sequence see Schults et al. 2010, 2013b) in a total volume of 25 μl. The PCRs were started by denaturation at 95 °C for 3 min, followed by 40 cycles of 95 °C for 10 s and 55 °C for 45 s. The PCR efficiency of all primer sets was assessed by the use of cDNA dilution curves and melt curves (55–95 °C). Data were analyzed by using MyiQ Software system (BioRad) and were expressed as relative gene expression (fold change) using the 2^−ΔΔCt^ method (McBrian et al. 2013). The Ct-value of the house-keeping gene *β*-*actin* and *GAPDH* was calculated for all samples and used as reference Ct-value. Since the expression Ct-value of *β*-*actin* is more close to our target genes, the final results are displayed as compared to house-keeping gene *β*-*actin*.

### Measurement of EROD activity

Ethoxyresorufin-*O*-deethylase (EROD) assay was used to assess the CYP1A1 activity in cells as described by Burke and Mayer (Burke and Mayer 1974) with modifications. Briefly, BEAS-2B and A549 cells were cultured in 96-well plate and treated with different pH and 1 μM B[a]P for 6, 24 and 48 h. After that, all cells were incubated with reaction mixtures which contained in the final volume (100 μl): Tris–HCl buffer (pH 7.4), 0.5 mM nicotinamide adenine dinucleotide phosphate (NADPH), 1.0 mg/ml Bovine Serum Albumin (BSA) and 5 μM 7-ethoxyresorufin (dissolved in DMSO). The reaction was initiated by adding NADPH. The formation of fluorescent resorufin was measured in a thermostated plate reader (Spectra max m2, MIDS, CA) at 37 °C and 535/590 nm excitation/emission wavelengths for 10 min. The results were calculated based on the resorufin standard curve and presented as RFU/min.

### Measurement of DNA incision activity

A modified comet assay was performed to assess the DNA repair activity as described by Langie et al. (2006), Azqueta and Collins (2013) and Azqueta et al. (2014). This method specifically measures the DNA damage recognition and DNA incision step of nucleotide excision repair. A549 cell suspensions were diluted 1:4 in 0.5% low melting point agarose and added to microscopic slides which were pre-coated with a layer of 1.5% normal melting point agarose and put at 4 °C for 45 min. Subsequently these cells were lysed overnight in cold (4 °C) lysis buffer (2.5 M NaCl, 0.1 M EDTA, 10 mM Tris, 0.25 M NaOH, pH 10, 1% Triton X-100 and 10% DMSO). After that, the cells were washed with cold phosphate-buffered saline (PBS) and exposed to either 1 μM BPDE (NCl Chemical Carcinogen Reference Standard Repository, Mid-west Research Institute, Kansas City, MO, USA) or DMSO for 30 min on ice.

Cell extracts were prepared from the cultured A549 and BEAS-2B cells with B[a]P and different pH media after 6, 24 and 48 h, and the method as developed by Langie et al. (2006) was used. Briefly, cells were trypsinized and diluted to a final concentration of 5 × 10^6^ cells/ml HBSS. Then, the cell suspensions were centrifuged at 14,000 rpm (20,800*g*) for 5 min, and the pellets were immediately resuspended in 50 μl buffer A (45 mM HEPES, 0.4 M KCl, 1 mM EDTA, 0.1 mM dithiothreitol, 10% glycerol, and adjusted to pH 7.8 using KOH) per 5 × 10^6^ cells. All aliquots were snap frozen in liquid nitrogen, and 15 μl of buffer B (1% Triton X-100 in buffer A) was added to each 50 μl aliquot. Then all the aliquots were centrifuged at 11,000 rpm at 4 °C for 5 min, and the supernatant was used as protein extract. The protein concentration in the supernatant was determined by the BIO-RAD DC Protein Assay Kit (BIO-RAD, Veenendaal, The Netherlands) using bovine serum albumin (BSA) as external standard. Finally, 1 mg/ml of protein extracts were used for repair assay and stored at −80 °C.

To assess the in vitro repair capacity, 50 μl of protein extracts were added onto either DMSO exposed or BPDE exposed gel-embedded nucleoids and incubated for 20 min at 37 °C. Subsequently, the slides were further processed according to the conventional comet assay as described by Collins (2004). The slides were stained with ethidium bromide (10 μg/ml) for 10 min, and the comets were visualized using a Zeiss Axioskop fluorescence microscope. All the samples were tested in two independent incubations within each single experiment in at least 2 independent experiments. For each slide, 50 cells were analyzed randomly using the Comet assay III software program (Perceptive Instruments, Haverhill, UK). The DNA incision activity of the cell extracts was calculated by the tail moment (median of the value) in the BPDE-modified nucleoids versus the DMSO-treated nucleoids. The final DNA incision activity was obtained according to the calculation made by Langie et al. (2006).

### ^32^P-postlabelling of B[a]P-DNA adducts

After cells were collected, DNA was isolated from cells using a phenol–chloroform–isoamylalcohol extraction procedure as described earlier by Schults et al. (2014). The DNA adduct level was determined according to the nuclease P1 enrichment technique as described by Reddy and Randerath (1987) with some modifications described by Godschalk et al. (1998). Two BPDE-DNA standards with known adduct levels (1 adduct/10^6^ and 1 adduct/10^7^ normal nucleotides) were used in all experiments and analyzed in parallel for quantification purposes. The quantification was conducted by using Phosphor-Imaging technology (Fujifilm FLA-3000, Rotterdam, The Netherlands).

### Histone protein H2AX phosphorylation assay (γH2AX)

DNA double-stranded breaks (DSBs) and blocked replication forks lead to the phosphorylation of histone protein H2AX on serine 139 (Muslimovic et al. 2008). We performed a γH2AX staining to investigate the DNA damage by the procedure as previously described by Mariotti et al. (2013) with some modifications. Briefly, after treatment with various pH and B[a]P at different time points, A549 cells were fixed in 2% formaldehyde for 20 min at room temperature. After fixation cells were permeabilized with 0.5% Triton X-100:PBS for 20 min at 4 °C and then blocked with 2% BSA and 0.5% Tween-20 in PBS. Cells were then stained with anti-γH2AX antibody (Upstate via Merck Millipore) overnight at 4 °C and the use anti-mouse Alexa fluor 488 secondary antibody (Life Technologies) for 1 h at 37 °C. Coverslips were mounted with *VECTASHIELD* ^*®*^ Mounting Medium containing DAPI, to counterstain cellular nuclei. γH2AX stained cells were scored manually by a digital fluorescent microscope with 100X objective, and the average number of positive cells was calculated from a minimum of 100 cells per dose/time point. Experimental data present the average of 4 independent experiments.

### Statistical analysis

Data are expressed as the mean ± standard error of the mean (SEM). GraphPad Prism 6 was used for statistical analysis. To assess the statistical significances between each incubation, a one-way analysis of variance test (ANOVA) with Bonferroni post hoc multiple comparison correction was used. To compare 2 groups, Student’s test was performed. Differences were considered to be statistically significant when *p* < 0.05. Correlations between the different variables were investigated using nonparametric spearman bivariate correlation analysis in the SPSS (v. 20.0) software package.

## Results

### Cell viability and cell proliferation rate

The results of the MTT assay were presented as a percentage of the results obtained for ‘physiological’ pH (i.e., pH of unmodified culture medium; pH 7.8). For A549 cells, MTT results demonstrated that although there is some toxicity at pH < 7 at various time points, these differences were not statistically significant when compared to their respective controls (Table 1). Furthermore, the results of the trypan blue exclusion assay for A549 cells indicated that the cell viability was above 93% at all time points.

**Table 1:** Cell viability test based on MTT assay and trypan blue after incubated A549 cells and BEAS-2B cells for 6, 24 and 48 h under combination of both 1 μM B[a]P with different pH (pH 7.8, 7, 6.5, 6 and 5.5) mediums

Methods	Time (h)	A549 cells					BEAS-2B cells				
		pH 7.8	pH 7	pH 6.5	pH 6	pH 5.5	pH 7.8	pH 7	pH 6.5	pH 6	pH 5.5
MTT assay	6	100 ± 1.2	92.1 ± 5.7	91.0 ± 3.9	93.4 ± 4.8	95.6 ± 5.2	100 ± 8.6	80.4 ± 7.8**	79.3 ± 5.5**	66.9 ± 3.2***	55.5 ± 3.8***
	24	100 ± 5.3	90.7 ± 8.9	95.5 ± 4.6	88.3 ± 3.6	87.7 ± 5.4	100 ± 17.2	56.6 ± 14.1***	53.3 ± 6.5***	40.8 ± 9.2***	31.5 ± 5.9***
	48	100 ± 5.1	83.5 ± 8.2	98.9 ± 1.2	95.5 ± 3.8	95.1 ± 3.3	100 ± 6.2	71.3 ± 1.9***	63.5 ± 6.8***	55.1 ± 7.1***	4.5 ± 0.3***
Trypan blue	6	98.0 ± 1.2	95.6 ± 3.0	95.1 ± 1.4	96.5 ± 0.8	93.7 ± 2.8	95 ± 0.1	97 ± 0.1	98 ± 0.1	96 ± 0.1	94.3 ± 1.2
	24	98.3 ± 1.2	97.7 ± 0.1	97.9 ± 1.5	95.5 ± 3.9	94.1 ± 1.0	96 ± 2	96.3 ± 1.5	92.3 ± 1.5*	59.7 ± 1.2***	54.7 ± 3.2***
	48	98.9 ± 0.7	97.7 ± 1.9	96.2 ± 2.8	94.4 ± 3.8	98.4 ± 1.3	96.3 ± 0.6	94.7 ± 1.2	93.3 ± 1.5*	65.3 ± 0.6***	34 ± 3***

Results of MTT are expressed as percentage of pH 7.8. Results of trypan blue are presented as survival rate (total live cells/total cells). All the value is presented as mean % ± SEM, *n* = 6

* *p* < 0.05

** *p* < 0.01

*** *p* < 0.001

MTT results were different for BEAS-2B cells (Table 1), essentially showing that BEAS-2B were more sensitive for low pH conditions: At *t* = 6 h, the viability significantly dropped at lower pH with approximately 20% at pH 7, to 45% at pH 5.5. The viability further declined in a pH-dependent manner after 24-h incubation. However, at *t* = 48 h, the viability for each condition improved by ca. 10–15%, except for pH 5.5. The results obtained by the trypan blue assay demonstrated a similar pattern as obtained with the MTT assay. At *t* = 6 h, the cell viability was above 94% for all conditions, but after 24-h incubation, exposure to pH < 6.5 resulted in significantly decreased cell viability. At pH ≥ 6,5, all samples showed a viability above 92%. Again, at *t* = 48 h, cell viability slightly recovered to 65% for pH 6, but further decreased to 44% for pH 5.5. Therefore, for BEAS-2B, only pH ≥ 6.5 were used for all subsequent experiments. pH did not influenced the cell proliferation rate.

### Intracellular and extracellular pH measurement

**Fig. 2:**
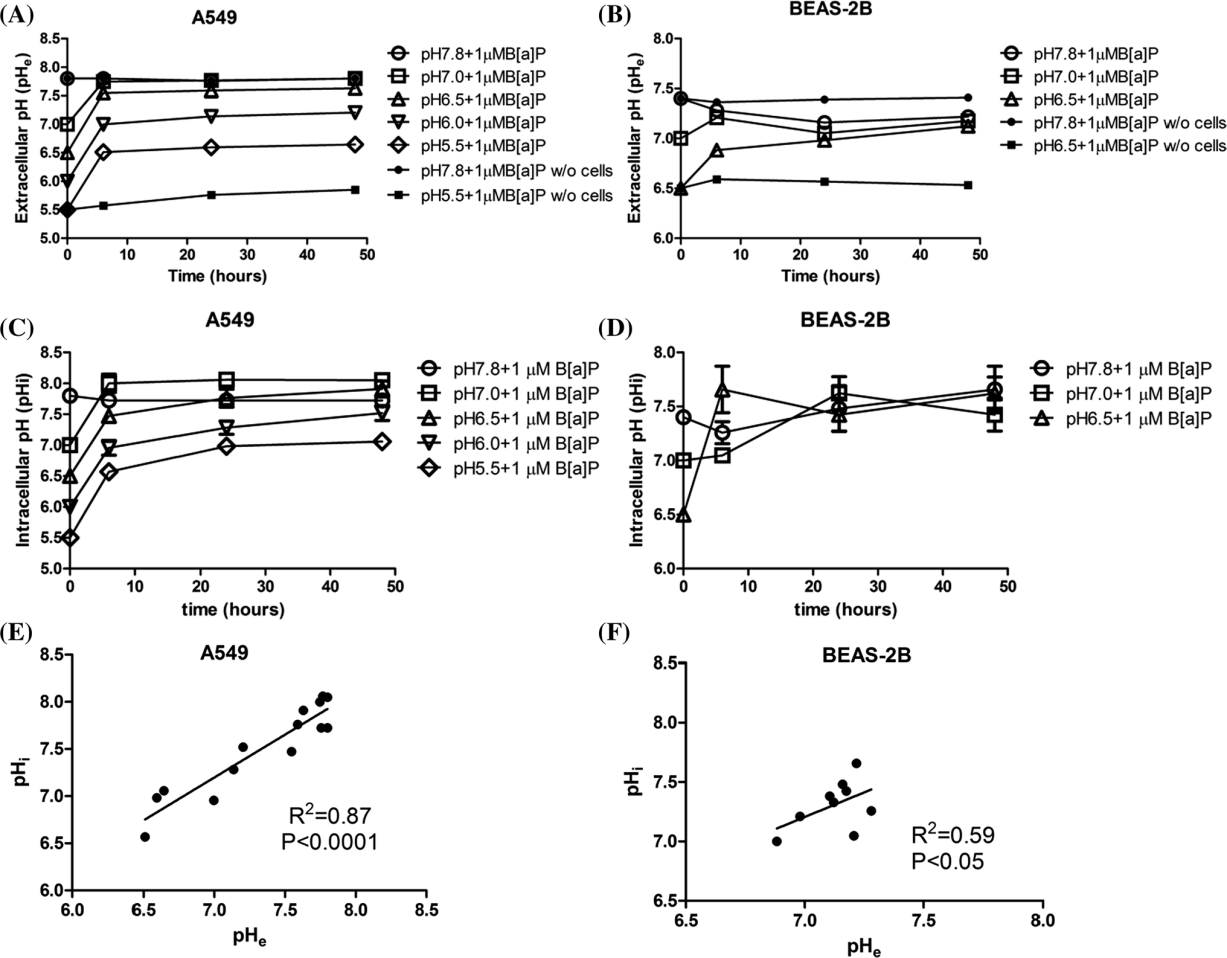
Measurements of pH_e_ and pH_i_ (mean ± SEM, *n* = 4 for pH_e_ and *n* = 8 for pH_i_). **a**, **b** Changes in extracellular pH_e_ in A549 and BEAS-2B cell culture medium (with and without cells) after 6, 24 and 48 h (w/o = without), respectively; **c**, **d** changes in intracellular pH_i_ in A549 and BEAS-2B cells after 6, 24 and 48 h, respectively; **e**, **f** correlation between pH_e_ and pH_i_ in A549 and BEAS-2B cells, respectively

The normal pH range for A549 cell culture medium is between 7.4 and 8.0 and for BEAS-2B between 7.2 and 7.8. After lowering the extracellular pH (pH_e_), the pH_e_ at least partially recovered after 6-h incubation in all A549 cell samples (Fig. 2a). The samples of pH_e_ 7 and pH_e_ 6.5 were raised again to a normal pH range (pH 7.8 and pH 7.6, respectively) after 6-h incubation, and samples maintained this pH_e_ after 48 h. However, for the relatively low pH_e_ samples (pH_e_ 6 and pH_e_ 5.5), the pHe was not fully restored to normal pH. After 6-h incubation, the pH_e_ for the samples that had an initial pH_e_ of 6 and 5.5, increased to pH 7.0 and pH 6.5, respectively. pH_e_ of these samples only slightly further restored after 48-h incubation to pH 7.2 and pH 6.6, respectively. Similar to A549 cells, the extracellular pH in all BEAS-2B cells sample was raised after 6-h incubation (Fig. 2b). The samples with pH_e_ 7.8 remained their extracellular pH, and the extracellular pH of samples with an initial pH_e_ 7 and pH_e_ 6.5 increased to pH 7.2 and pH 6.9, respectively. At *t* = 24 h, the extracellular pH of pH_e_ 6.5 partially restored to pH 7. After 48-h incubation, all samples’ pH reached pH 7.1–7.2. Furthermore, in order to confirm that the restored pH is an effect of the presence of cells, two pH media (RPMI pH 7.8 and pH 5.5; DMEM pH 7.8 and pH 6.5) without cells were cultured under the same conditions. Figure 2a, b shows that the pH of the medium is not able to restore without the presence of cells.

The intracellular pH (pH_i_) of A549 cells changed in time in accordance with their extracellular pH, (Fig. 2c) and consequently, pH_e_ and pH_i_ correlated very well (*R* ^2^ = 0.87, *p* < 0.0001, Fig. 2e). With increasing incubation time, the pH_i_ restored to the ‘physiological’ pH range (pH 7.4–8.0), but only in those samples in which the initial pH_e_ at *t* = 0 was above 6.5. For instance, the pH_i_ of the samples with an initial pH_e_ of 6.5 increased to pH_i_ 7.4 and pH_i_ 7.6 at, respectively, *t* = 6 and 24 h. However, at low initial pH (pH_e_ 6 and 5.5), the pH_i_ remained lower than in untreated cells with pH_i_ 7.3 and pH_i_ 7.0, respectively. At *t* = 48 h, the recovery of pH_i_ of the lowest pH_e_ sample (pH 5.5) only reached to pH_i_ 7.0.

For BEAS-2B cells, the results were essentially similar; the pH_i_ changes in time generally in line with the respective pH_e_ with a good correlation between both parameters (Fig. 2d, f, *R* ^2^ = 0.59, *p* < 0.05). After 6 h incubations, all samples’ pH_i_ restored to pH above 7 (pH 7.3 for pH_e_ 7.8, pH 7 for both pH_e_ 7 and pH_e_ 6.5, respectively). At *t* = 24 and 48 h, the pH_i_ in all samples were within the BEAS-2B ‘physiological’ pH range (7.2–7.8).

### B[a]P and B[a]P metabolites

**Fig. 3:**
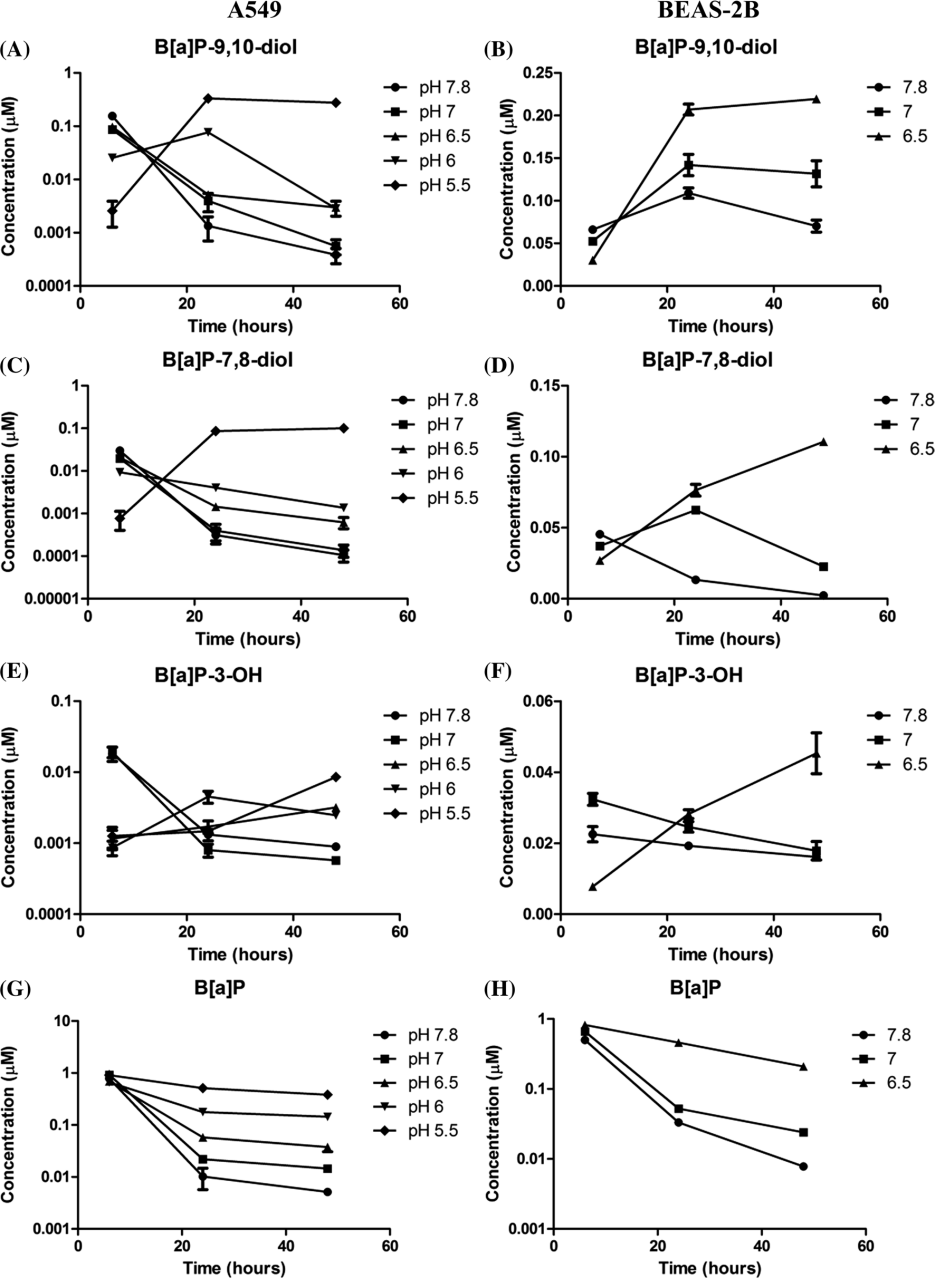
A549 and BEAS-2B cells were treated with 1 µM B[a]P and incubated at the indicated pH_e_ for 6, 24 and 48 h. The *left column* represents data from A549 cells and the right column from BEAS-2B cells. The extracellular concentrations of B[a]P-9,10-diol (**a**, **b**); B[a]P-7,8-diol (**c**, **d**); B[a]P-3-OH (**e**, **f**); and B[a]P (**g**, **h**) were measured at the indicated time points. Data are presented as mean ± SEM, *n* = 4 independent experiment. The results of statistical analysis are included in supplemental Table 1

Three important extracellular B[a]P metabolites, B[a]P-9,10-dihydrodiol (B[a]P-9,10-diol), B[a]P-7,8-dihydrodiol (B[a]P-7,8-diol) and 3-hydroxy-B[a]P (B[a]P-3-OH) and unmetabolized B[a]P were measured in order to determine how changes in pH affect the metabolism of B[a]P (Fig. 3, for detail metabolites data see Supplement Table 1). In A549 cell with decreased pH_e_, the extracellular B[a]P-7,8-diol levels after 6 h of incubation were significantly lower compared to the normal pH (pH 7.8) with decreases of ~34% (pH 7, *p* < 0.001), ~31% (pH 6.5, *p* < 0.01), ~69% (pH 6, *p* < 0.001) and ~97% (pH 5.5, *p* < 0.001), respectively. After 24 h, the concentration of B[a]P-7,8-diol was still lower in those samples whose pH was decreased, except at the lowest pH_e_ (pH 5.5). At *t* = 48 h, the B[a]P-7,8-diol levels in samples with an initial pH_e_ 5.5 kept increasing to 101 nM, which was 1000-fold higher than its concentration at pH 7.8. On the other hand, the B[a]P-7,8-diol levels in the other samples declined over time to 1.4–0.1 nM after 48-h incubation; the concentration at *t* = 48 h is pH dependent. Extracellular concentrations of B[a]P-9,10-diol and B[a]P-3-OH (Fig. 3a, e) displayed a similar pattern as the concentration of B[a]P-7,8-diol. The extracellular metabolite concentrations were lower at pH_e_ 5.5 at *t* = 6 h. However, at *t* = 48 h, the concentration of B[a]P-9,10-diol and B[a]P-3-OH at pH_e_ 5.5 was 336-fold and ninefold higher than the concentrations observed at pH_e_ 7.8, respectively.

The extracellular metabolites level (B[a]P-9,10-diol, B[a]P-7,8-diol and B[a]P-3-OH) in BEAS-2B cells displayed a similar patterns as in A549 cells, but the effects were already seen at a non-cytotoxic extracellular pH of 6.5 (Fig. 3). After 6-h incubation, the concentration of B[a]P-7,8-diol in pH 7 (*p* < 0.05) and pH 6.5 (*p* < 0.001) was significantly lower than at pH_e_ 7.8, respectively. At *t* = 24 h, the level of B[a]P-7,8-diol in pH_e_ 7.8 decreased about 69% when compared to its level at *t* = 6, whereas the concentration of B[a]P-7,8-diol in the samples with pH_e_ 7 and pH_e_ 6.5 increased approximately 1.7-fold and 3.4-fold, respectively. At *t* = 48 h, the B[a]P-7,8-diol levels at pH 7 (*p* < 0.001) decreased to 22.7 nM, but was still significantly higher than at pH_e_ 7.8. The B[a]P-7,8-diol concentration at pH_e_ 6.5 kept increasing in time to 111 nM and was ultimately 48-fold higher than at pH_e_ 7.8. Extracellular B[a]P-9,10-diol and B[a]P-3-OH (Fig. 3b, f) levels were initially significantly lower at pH_e_ 6.5 compared to pH_e_ 7.8 at *t* = 6 h, ≈54% (*p* < 0.001) and ≈65% (*p* < 0.001), respectively. However, at *t* = 24 h, concentrations of both metabolites further increased at pH_e_ 6.5 but decreased in pH_e_ 7.8. Consequently, at *t* = 48 h, both metabolites were 3.1-fold and 2.8-fold higher at pH_e_ 6.5 than at pH_e_ 7.8, respectively.

The extracellular kinetics of unmetabolized B[a]P is indicative for the rate of metabolism of B[a]P under each pH condition (Fig. 3g, h). In A549 cells, the level of metabolized B[a]P in time was reduced with decreasing pH_e_, eventually reaching a > 50-fold difference when pH_e_ 5.5 was compared with pH_e_ 7.8 (*p* < 0.001). The concentration of unmetabolized B[a]P was pH dependent (*R* ^2^ = 0.71, *p* = 0.074). In BEAS-2B cells, the level of extracellular unmetabolized B[a]P showed a similar pattern as for A549 cells; the concentration of unmetabolized B[a]P was significantly higher at low pH than at neutral pH in a pH-dependent manner (*R* ^2^ = 0.68, *p* = 0.0009).

### CYP1A1 and CYP1B1 gene expression and EROD activity

**Fig. 4:**
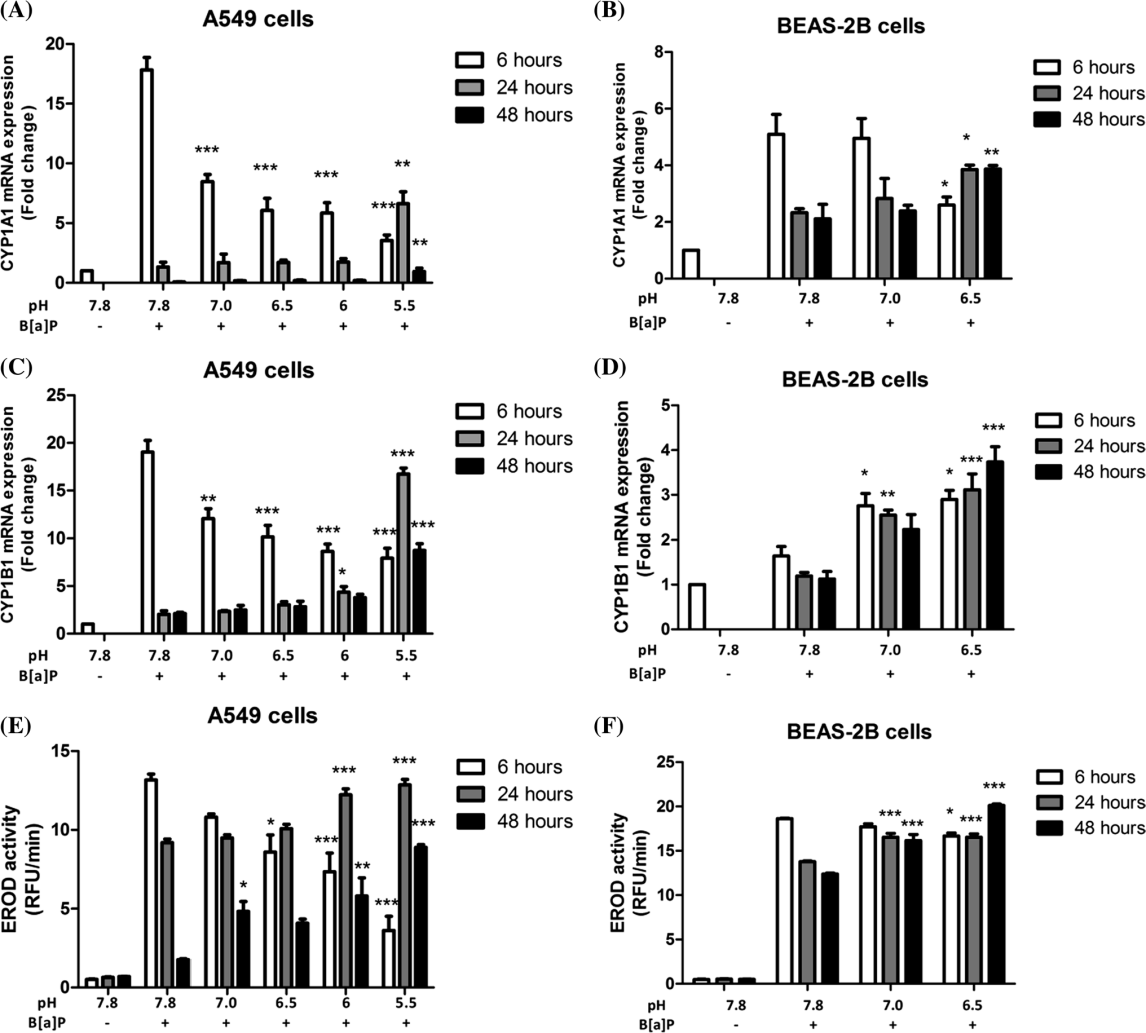
A549 and BEAS-2B cells were treated with 1 µM B[a]P and incubated at the indicated pH_e_ for 6, 24 and 48 h. *CYP1A1* mRNA expression level was assessed in A549 cells (**a**) and BEAS-2B cells (**b**) by qRT-PCR. *CYP1B1* mRNA expression level was assessed in A549 cells (**c**) and BEAS-2B cells (**d**) by qRT-PCR. All data were normalized with the house-keeping gene (*β*-*actin*) and compared to controls (A549 cells and BEAS-2B cells incubated with DMSO for 6 h) and expressed as mean ± SEM, *n* = 4; CYP450 s activity (CYP1A1 activity) was measured by an EROD assay in A549 cells (**e**) and BEAS-2B cells (**f**). Data are presented as relative fluorescence units/min (RFU/min) and expressed as mean ± SEM, *n* = 5. Statistical comparison was performed between each pH conditions (pH 7, 6.5 6 and 5.5) with pH 7.8 at each time point. *p < 0.05; **p < 0.01 and ***p < 0.001

To determine whether the activity of the main cytochrome P450 enzyme that is involved in B[a]P metabolism was altered by decreased pH in cells, we assessed the mRNA expression of *CYP1A1* and *CYP1B1* and EROD activity as a indicator of CYP1A1 activity for both A549 (Fig. 4a, c, e) and BEAS-2B (Fig. 4b, d, f) cells. In A549 cells (Fig. 4a), *CYP1A1* expression decreased to 48% (pH 7, *p* = 0.0002), 34% (pH 6.5, *p* = 0.0002), 33% (pH 6, *p* = 0.0001) and 20% (pH 5.5, *p* < 0.0001) compared to pH 7.8 when exposed to B[a]P for 6 h, respectively. However, the pH-dependent trend converted after 24 h in such a way that *CYP1A1* expression was now up-regulated as the initial pH decreased. When compared to pH 7.8 with B[a]P at *t* = 24 h, the *CYP1A1* expression at pH 7, pH 6.5, pH 6 and pH 5.5 was 1.3-fold, 1.3-fold, 1.3-fold and fivefold (*p* = 0.0025) increased, respectively. This trend remained after 48-h incubation, and the lowest pH (pH 5.5) showed a significant induction of *CYP1A1* (12.9-fold, *p* < 0.001) compared to pH 7.8 with B[a]P. For the mRNA expression of *CYP1A1* in BEAS-2B cells, a similar pattern as described for A549 cells was observed but less pronounced (Fig. 4b). At *t* = 48 h pH_e_ = 6.5, the *CYP1A1* expression was decreased in A549 cells but kept increasing in BEAS-2B cells.

Figure 4c, d indicates the changes of *CYP1B1* gene expression after incubation with B[a]P under various pH conditions for 6, 24 and 48 h in both A549 and BEAS-2B cells, respectively. The *CYP1B1* mRNA expression changes in A549 cells were similar to the changes seen for *CYP1A1* gene expression. At *t* = 6 h, an 19-fold increasing of *CYP1B1* mRNA expression was observed after treatment with 1 μM B[a]P at pH 7.8. Meanwhile, a significantly pH-dependent decrease in *CYP1B1* gene expression was found when compared to pH 7.8, and the *CYP1B1* mRNA level at the lowest pH_e_ (pH_e_ 5.5) was 42% of pH 7.8 (Fig. 4c). After 24-h incubation, the *CYP1B1* mRNA level decreased at neutral pH. Although the *CYP1B1* mRNA expression also decreased for the rest of pH samples (except pH 5.5), the trend was a pH-dependent increase when compared to pH 7.8. The *CYP1B1* mRNA level in samples with initial pH_e_ 5.5 was about eightfold higher than at pH 7.8 at *t* = 24 h. Additionally at *t* = 48 h, the *CYP1B1* mRNA levels were back to basal level in most samples, except for samples with pH 5.5, in which the expression remained 8.7-fold increased compared at pH 7.8. For BEAS-2B cell, the expression of *CYP1B1* showed a slightly different trend when compared to *CYP1A1* gene expression at *t* = 6 h. The *CYP1B1* gene expression at pH 7 and pH 6.5 demonstrated 1.7-fold and 1.8-fold higher levels than at pH 7.8, respectively (Fig. 4d). At *t* = 24 h and *t* = 48 h, the *CYP1B1* mRNA levels at both pH 7 and pH 6.5 remained significantly higher than at pH 7.8, eventually, pH 6.5 displayed 3.3-fold higher *CYP1B1* gene expression after 48-h incubation when compared to samples that were incubated at pH 7.8.

The EROD activity as an indicator of CYP1A1 activity was measured under different pH conditions for 6, 24 and 48 h for both A549 and BEAS-2B cells (Fig. 4e, f). The results of the EROD activity paralleled the results of *CYP1A1* expression for both cell lines. In A549 cells at *t* = 6 h, the low pH treatment resulted in significantly lower EROD activity (pH 6.5, *p* < 0.05; pH 6, *p* < 0.001 and pH 5.5, *p* < 0.001, respectively) when compared to pH 7.8, but at *t* = 24 h, the EROD activity in low pH and B[a]P-treated samples showed significantly higher activity (pH 6, *p* < 0.001 and pH 5.5, *p* < 0.001) when compared to pH 7.8. After 48-h incubation, although the EROD activity was decreased in all samples, the activity in the low pH_e_-treated samples remained higher than at pH 7.8 (see Fig. 4c, pH 6, *p* < 0.01 and pH 5.5, *p* < 0.001). The EROD activity in BEAS-2B cells displayed similar trends and corresponded to the *CYP1A1* gene expression.

### pH dependency of DNA incision activity

**Fig. 5:**
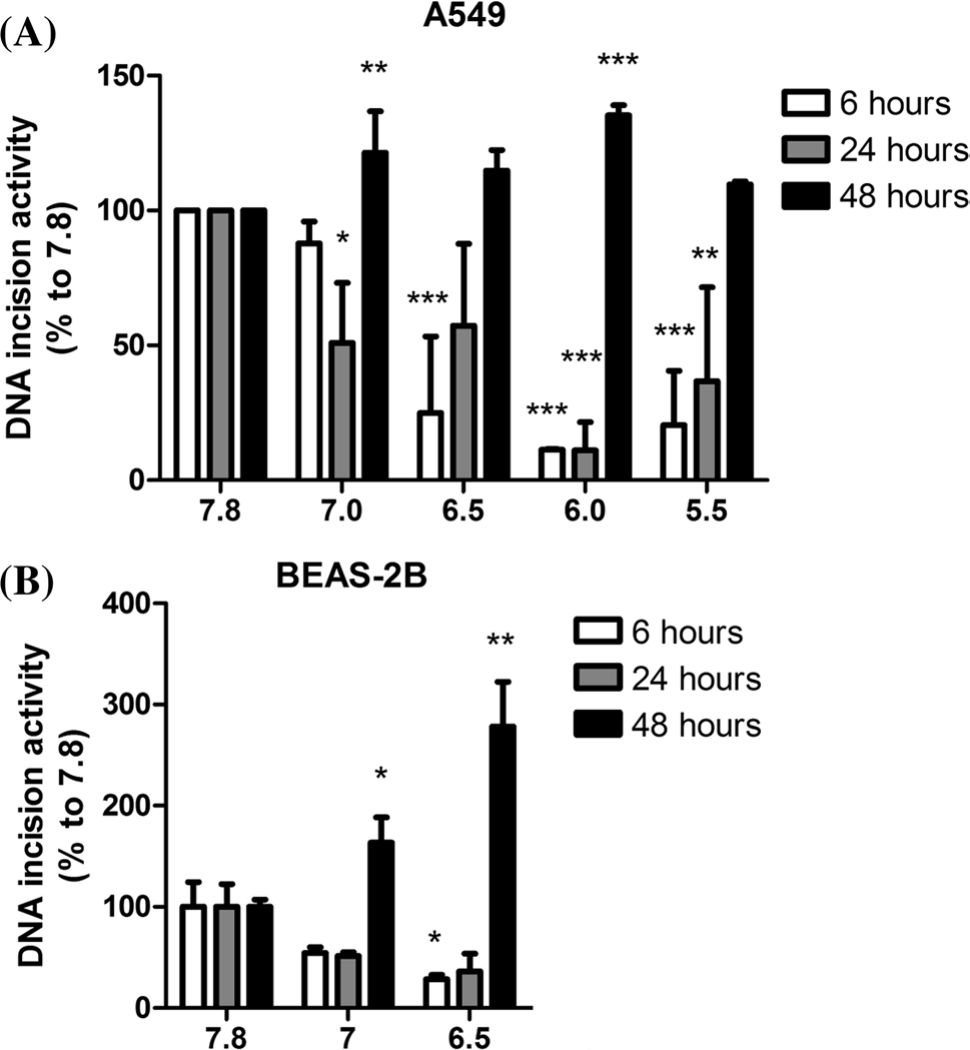
DNA incision activity measured by a modified comet assay. Nuclei were treated with BPDE, and cell extracts were prepared from cells incubated at different pH and B[a]P for variable times. These extracts were then used to treat the BPDE-damaged nuclei for 20 min. The DNA incision activity of the extract was determined by the comet assay. DNA incision activity was calculated as percentage of the values observed in pH 7.8 samples (pH 7.8 with B[a]P at 6, 24 and 48 h) (mean ± SEM, *n* = 4). All raw data (Tail Moment and Tail Intensity) for both cell lines will be presented in Supplemental Table 2; **a** A549 cells; **b** BEAS-2B cells; statistical comparison was performed between each pH conditions with pH 7.8 with BaP only at each time point. *p < 0.05; **p < 0.01 and ***p < 0.001

DNA adduct levels are the net effect of DNA adduct formation and removal by DNA repair. Therefore, the impact of acidic pH on DNA incision activity in both cell lines (A549 and BEAS-2B) was assessed (Fig. 5). In A549 cells, at *t* = 6 h, a markedly reduced DNA incision activity was observed at lowered pH_e_.; approximately 25% (pH 6.5, *p* < 0.001), 11% (pH 6, *p* < 0.001) and 20% (pH 5.5, *p* < 0.001) of DNA incision activity remained when compared to pH 7.8. After 24-h incubation, the DNA incision activity in the majority of samples (except pH 7) partly recovered, but at low pH_e_, the DNA incision activity remained lower than at pH 7.8 (pH 6, *p* < 0.001 and pH 5.5, *p* < 0.01). Interestingly, at *t* = 48 h, the DNA incision activity fully recovered to more than 100% compared to pH 7.8 for all the samples, including pH 7 (121%, *p* < 0.01), pH 6.5 (115%, *p* = 0.065), pH 6 (135%, *p* < 0.001) and pH 5.5 (110%, *p* = 0.062). For BEAS-2B cells, although there were only three pH conditions (pH 7.8, pH 7 and pH 6.5) tested, a similar trend as for A549 cells was observed (Fig. 5b). At *t* = 6, when compared to pH 7.8, a strong decreased DNA incision activity was found at pH 7 (decrease of 45%, *p* = 0.14) and pH 6.5 (decrease of 77%, *p* < 0.05). After 24-h incubation, for both pH 7 and pH 6.5, the DNA incision activity was slightly raised but remained lower than at pH 7.8 (but not statistically significant). At *t* = 48 h, for both pH 7 and pH 6.5, the DNA incision activity was significantly increased to 1.6-fold (*p* < 0.05) and 2.8-fold (*p* < 0.01) higher than at pH 7.8, respectively.

### pH-dependent levels of B[a]P-DNA adducts

**Fig. 6:**
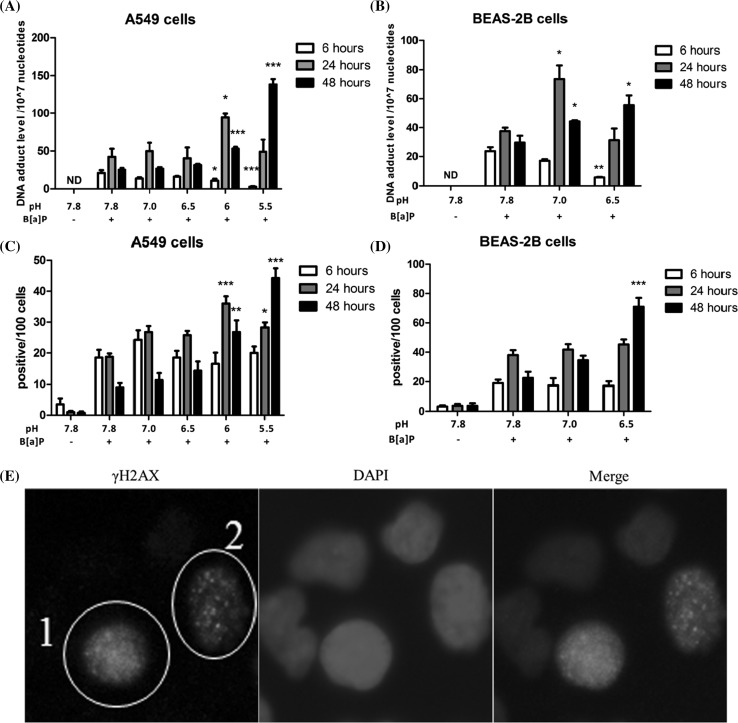
A549 and BEAS-2B cells were treated with 1 µM B[a]P and incubated at the indicated pH_e_ for 6, 24 and 48 h. **a** DNA adduct levels in A549 cells. **b** DNA adduct levels in BEAS-2B cells. DNA was isolated and analyzed by ^32^P-postlabeling. Data are presented as mean DNA adduct level per 10^7^ nucleotides ± SEM (*n* = 4). *ND* not detected. **c** DNA damage in A549 cells measured by *γ*H2AX staining. **d** DNA damage in BEAS-2B cells measured by *γ*H2AX staining. Data are expressed as positive cells per 100 cells (mean ± SEM, *n* = 4). **e** Example of positive cells under 100X objective using a digital fluorescent microscope (including *γ*H2AX staining, DAPI and merge). *Yellow circle* of 1 and 2 represent cells that were counted as positive; statistical comparison was performed between each pH conditions with pH 7.8 with B[a]P at each time point. *p < 0.05; **p < 0.01 and ***p < 0.001 (color figure online)

As shown in Fig. 6a, incubation of A549 cells with different pH_e_ in combination with B[a]P initially resulted in significantly lower DNA adduct levels as the pH_e_ decreased (pH 6, *p* < 0.05 and pH 5.5, *p* < 0.001). After 24 h of incubation, the B[a]P-DNA adduct levels were increased for all samples, but DNA adduct levels increased predominantly in the samples that were treated at pH 6 (*p* < 0.05). At *t* = 48 h, the DNA adduct levels decreased in all samples, except at pH 5.5. After 48 h at pH 5.5, DNA adduct levels were 138 adducts per 10^7^ nucleotides which is 5.5-fold higher than the adduct levels observed for samples that were incubated at pH_e_ 7.8 (*p* < 0.001).

A pH dependency of B[a]P-induced DNA adduct levels was also found for BEAS-2B cells (Fig. 6b). At *t* = 6 h, 24 adducts per 10^7^ nucleotides were reached at pH 7.8. Samples with initial pHe 7 and pHe 6.5 contained 28% and 76% (*p* < 0.01) lower DNA adduct levels than at pHe 7.8, respectively. However, after 24 h of incubation, the B[a]P-DNA adduct levels increased for all samples and the DNA adduct level at pH 7 was 74 adducts per 10^7^ nucleotides which was twofold (*p* < 0.05) higher than at pHe 7.8. At *t* = 48 h, the DNA adduct levels decreased at pH 7.8 and pH 7, but not for pH 6.5. Eventually, the DNA adduct levels for pH 7 and pH 6.5 were 1.5-fold (*p* < 0.05) and 1.9-fold higher (*p* < 0.05) than in samples in which the initial pHe was 7.8 after 48-h incubation, respectively.

### Histone protein H2AX phosphorylation (γH2AX)

To determine the overall DNA damage after exposure of A549 and BEAS-2B cell at different pH’s in combination with B[a]P, we measured the phosphorylation of histone protein H2AX under each condition (Fig. 6). At pH 7.8, exposure of both cell lines (A549 and BEAS-2B cells) to B[a]P significantly induced DNA damage after 6, 24 and 48 h. In A549 cells (Fig. 6c), when comparing to B[a]P-exposed samples at pH 7.8, there was no significant difference at *t* = 6 h for samples that were incubated at lower pH. However, at *t* = 24 h, the DNA damage was enhanced for all lowered pH conditions; pH 6 (*p* < 0.001) and pH 5.5 (*p* < 0.05) showed a 1.9- and 1.5-fold higher percentage of γH2AX stained cells than at pH 7.8, respectively. At *t* = 48 h, the DNA damage level decreased in all samples, except for samples with pH 5.5. Moreover, DNA damage at pH 6 (*p* < 0.01, threefold) and pH 5.5 (*p* < 0.001, 4.9-fold) was still significantly higher after 48 h of incubation when compared to levels of γH2AX staining at pH 7.8.

Interestingly, again similar results were also found for BEAS-2B cells (Fig. 6d). After 6- and 24-h incubation, the DNA damage increased in all samples and there was no significant difference between any pH condition and pH 7.8. However, at *t* = 48 h, the number of positive cells at pH 7.8 and pH 7 decreased, whereas the positive cells increased at pH 6.5 to a 3.2-fold (*p* < 0.01) higher level when compared to samples that were incubated at pH 7.8.

### Correlation between each variable (B[a]P,7-8-diol, CYP1A1/CYP1B1 mRNA expression, EROD activity, DNA incision activity and DNA adduct)

After measuring different types of variables related to B[a]P-related metabolism or B[a]P-induced DNA damage, correlations between the variables were assessed for both cell lines. Most parameters related to B[a]P metabolism correlated with each other in both cell lines, because they are involved in the same pathway. For instance, in A549 cells (Supplemental Table 3), the extracellular concentration of B[a]P-7,8-diol correlated significantly with *CYP1A1* mRNA expression (*r* = 0.704, *p* < 0.01), *CYP1B1* mRNA expression (*r* = 0.914, *p* < 0.001), EROD activity (*r* = 0.607, *p* < 0.05) and B[a]P-induced DNA adduct level (*r* = 0.555, *p* < 0.05). None of variable showed a good correlation with DNA incision activity. In addition, only B[a]P-7,8-diol, the precursor of BPDE, demonstrated good correlation with BPDE-DNA adduct level.

In BEAS-2B cells (Supplemental Table 4), the extracellular concentration of B[a]P-7,8-diol showed significant correlations with *CYP1A1* mRNA expression (*r* = 0.750, *p* < 0.01), *CYP1B1* mRNA expression (*r* = 0.800, *p* < 0.01), EROD activity (*r* = 0.733, *p* < 0.05) and B[a]P-induced DNA adduct level (*r* = 0.717, *p* < 0.05). Similar to A549 cells, none of variables gave significant correlations with DNA incision activity and only B[a]P-7,8-diol displayed a significant correlation with DNA adduct level.

## Discussion

A low extracellular pH can occur for instance in tumors (Vaupel et al. 1989), but also at sites of inflammation (Lardner 2001) and an acidic microenvironment may drive the initiation and progression phase of the carcinogenic process. We found in the present study that a change in extracellular pH (pH_e_) leads to a corresponding change in intracellular pH (pH_i_), which affect xenobiotic activation and deactivation processes. Cells try to restore the normal situation by increasing the pH via H+ ATPases and H+-pumps (van Adelsberg and Al-Awqati 1986), and indeed we found that 48 h after starting acidic in vitro incubations, most of the samples’ pH (pH_e_ as well as pH_i_) were back at normal levels (pH 7.8). Interestingly, we noticed that pH_i_ was always slightly higher than pH_e_ and this phenomenon was also observed by McBrian et al. (2013) and Justus et al. (2013). For samples that were exposed to relatively low pH (pH 6 and pH 5.5), the pH_e_ and pH_i_ did not fully recover and maximally reached to pH 7.2 and pH 6.5 after 48-h incubation, respectively.

It has been reported that acidic pH reduces cell density and cell viability, which may be cell-type dependent. For example, incubation of neurons and glial cells under pH 5.0 for 1 h decreased the number of viable cells to <50% (Nedergaard et al. 1991), and mouse epithelial cells demonstrated a significantly decreased cell viability in an acidic dose-dependent manner after 1-h incubation (Block et al. 2010) and also Chinese hamster ovary (CHO) cells exposed to low pH showed cytotoxicity in a time- and pH-dependent manner (Rotin et al. 1986). In our study, an acidic pH of <6.5 similarly led to decreased viability in human bronchial epithelial cells (BEAS-2B) (Table 1). However, most tumor cell types seem to be able to resist acidic environments quite well. In a previous study, we showed that a human liver hepatocellular carcinoma cell line (HepG2) could survive more than 48 h at pH 5.5 (Shi et al. 2015). Moreover, there was no noticeable drop in cell viability by low pH in cancer type cells as LlA2 ascites cells (at pH 6.4–7.2) or MCF-7 cells (Lee et al. 2003). In the our study, we additionally cultured A549 cells (human lung carcinoma cell) under various pH’s and the cell viability was always above 80% even after 48-h incubation at pH 5.5, indicating a relative resistance to acidic conditions. BEAS-2B cell was more sensitive for acidic conditions, and pH’s above 6.5 had to be used for culturing BEAS-2B cells to avoid cytotoxicity. We initially used the MTT test to check for cell viability, which actually measures the activity of mitochondrial succinate dehydrogenase, and the activity of that enzyme may be pH dependent (Plumb et al. 1989). Thus, trypan blue exclusion was used as an alternative method, and the results showed that >90% of A549 cells were viable after incubation at various pH for up to 48 h. In addition, an acidic microenvironment is associated with promotion of cell growth (Kato et al. 2013). In our study, we indeed observed a growth promoting effect on A549 cells of low pH (i.e., pH 6 and pH 5.5) but the difference in the number of cells at the end of the incubation was not statistically significant.

Pulmonary inflammation is often induced by inhalation of particles, from smoking or other environmental sources and can locally reduce the pH in inflamed tissue. Vice versa, lowering extracellular pH can increase the inflammatory response and can induce hypoxia, which results in the formation of reactive oxygen species (ROS) and subsequent DNA damage (Riemann et al. 2011; Rotin et al. 1986). However, inflammation induced by exposures is often in combination with ubiquitous environmental contaminants such as polycyclic aromatic hydrocarbons (PAH). We previously showed that the presence of inflammation may change the way in which cells metabolize the model PAH, benzo[a]pyrene (B[a]P) (Borm et al. 1997; Schults et al. 2014). Therefore, we investigated whether a low pH affected B[a]P metabolism, DNA adduct formation and removal, and DNA damage. We investigated the expression and activity of *CYP1A1* and *CYP1B1*, which is important for B[a]P metabolism. We found a lower initial expression of both *CYP1A1* and *CYP1B1* and EROD activity at acidic pH conditions (pH 5.5–6) than at normal pH (pH 6.5–7.8). However, while the pH restored, both *CYP1* expression and its activity significantly increased for up to 48 h at conditions of lowered pH. On the other hand, at neutral pH both expression and EROD activity of CYP decreased. As a result, the metabolism of B[a]P was initially high at neutral pH and was inhibited at low pH. Inhibition of B[a]P metabolism resulted in accumulation of unmetabolized B[a]P which keeps triggering the aryl hydrocarbon receptor (AhR) that up-regulates various B[a]P metabolizing enzymes (Schults et al. 2013b), which all have their own optimal pH range. For example, the phase I enzyme cytochrome P450 works at pH 5.9–8.5, and its optimal pH is between 7.4 and 7.8 (Axarli et al. 2010; Sen and Arinc 1998). Moreover, also UDP-glucuronosyltransferase (UGTs) isozymes, which are important phase II enzymes for detoxification of B[a]P metabolites, exhibit a broad pH range from 5.5 to 8.5 in which they can function (Basu et al. 2004). The UGTs superfamily is large, and therefore, UGT’s are active over a very wide pH range, and each UGT has its own optimal pH (Luque et al. 2002). Since B[a]P metabolizing enzymes have different relative activities at different pH, it can be expected that B[a]P metabolism is significantly affected by pH. Indeed, at *t* = 48 h, B[a]P and its metabolites were close to non-detectable in samples that were incubated at a neutral pH range (pH 6.5–7.8) in A549 cells and at pH 7.8 in BEAS-2B cells, whereas B[a]P and its metabolites remained high in low pH samples with a notable increase in B[a]P-7,8-diol, which is the precursor of the pro-mutagenic BPDE. Therefore, it seems that low pH results in delayed B[a]P metabolism which we previously observed under hypoxic conditions (Schults et al. 2014) and in the presence of beta-glucuronidase (Shi et al. 2015).

Most of the DNA damage and BPDE-DNA adducts will be removed by nucleotide excision repair (NER) in which DNA incision is a rate limiting step (Lindahl and Wood 1999). Therefore, in the present study we also investigated whether acidic pH inhibits DNA incision activity. After incubation of A549 and BEAS-2B cells at acidic pH, the DNA incision activity was inhibited by approximately 70–90%, but repair activity recovered when pH restored to close to neutral levels. We previously reported that hypochlorous acid (HOCl) dose dependently inhibited DNA incision activity (Gungor et al. 2007; Whiteman et al. 2002). We attributed this decrease in DNA incision activity to the release of inflammation-related MPO (myeloperoxidase), because the in vitro inhibition of MPO restored DNA incision activity. In later in vivo experiments, the effect appeared not to be MPO dependent, because inhibition of MPO release or knock out of MPO could not restore DNA incision activity during inflammation in vivo (Gungor et al. 2010). Therefore, we now suggest that the effect on DNA incision activity was not specifically related to HOCl, but can better be explained by an acidic pH.

The altered metabolism of B[a]P ultimately resulted in higher levels of DNA damage under conditions of low pH for both cell lines. DNA damage is a continuous process/combinational effect of accumulation of damage (activation) and elimination by DNA repair (detoxification) (Manière et al. 2005). At *t* = 6, the B[a]P-induced DNA damage is significantly lower at low pH when compared to neutral pH (pH 7.8). This is due to reduced metabolism of B[a]P under acidic condition that leads to lower amount of B[a]P-7,8-diol formation, and subsequently low BPDE lesions were observed at low pH samples at 6 h. Although the DNA repair was significantly inhibited by acidic pH at 6 h, the effect of DNA repair in DNA adduct removal is less strong at low pH samples than at pH 7.8. After 24-h incubation, the DNA adducts were increased for all samples since the intracellular pH and metabolism were restored. However, the metabolism in the lowest pH samples (e.g., pH 5.5 for A549 cells and pH 6.5 for BEAS-2B cells) stayed low. Meanwhile, the DNA incision activity also remained low for low pH samples. Therefore, the highest DNA adduct level was reached by the sample which is not under extreme acidic conditions (e.g., pH 6 for A549 cells and pH 7 for BEAS-2B cells). At *t* = 48 h, the intracellular pH completely restored for all samples as well as the metabolism and DNA repair. Hence, we observed a decrease in BPDE-related lesions for all samples at 48 h (except for pH 5.5 for A549 cells and pH 6.5 for BEAS-2B cells).

Correlation analysis confirmed the interrelationship between the various metabolic parameters. For examples, CYP1B1 was reported to be involved in the metabolic activation of B[a]P (Schults et al. 2013a; Smerdova et al. 2013). Therefore, we observed that the extracellular B[a]P-7,8-diol concentration showed a good correlation with *CYP1B1* mRNA gene expression. Furthermore, *CYP1A1* mRNA gene expression gave a better correlation with EROD activity than *CYP1B1* for both cell line (van Duursen et al. 2005). Moreover, we also observed that only the B[a]P-7,8-diol concentrations demonstrated a good correlation with DNA adducts level in both cell line. This is due to the fact that DNA adducts measured in the current study were most probably related to BPDE and B[a]P-7,8-diol is the precursor to the BPDE (Fang et al. 2002; Moserová et al. 2009).

Because of this interrelation of many of the parameters that we assessed, investigating the role of acidic pH in B[a]P metabolism is not straightforward and therefore our study has some limitations. Firstly, lowering extracellular pH will affect not only the intra- and extracellular pH balance, but also all kind of cellular enzyme activities and downstream pathways. For instance, acidic pH can activate inflammation-related pathway, including activator protein 1 (AP-1), MAPK and nuclear factor kappa-light-chain-enhancer of activated B cells (NF-κB) pathways which result in an induction of a series of genes, such as tumor necrosis factor α (TNF-α), extracellular signal-regulated kinases (ERK1/2), isoform of nitric oxide synthase (iNOS), and p38 (Bellocq et al. 1998; Kato et al. 2013; Riemann et al. 2011). In addition, the induction of TNF-α further increases genotoxic effect of B[a]P via for instance up-regulation of p38 and CYP1B1 (Umannova et al. 2008, 2011). Besides these, acidic pH also causes hypoxia like reactions, oxidative stress and ROS formation which makes the results of current study more complicated to explain from a mechanistic point of view (Chiche et al. 2010; Kato et al. 2013; Maurer et al. 2005; Riemann et al. 2011). For example, we observed a pH-dependent DNA incision activity in the present study; however, it might be the combined result of ROS inhibiting DNA repair via targeting at the thiol groups of DNA repair enzymes (Bertin and Averbeck 2006; Klaunig et al. 2010) or because of changes in GSH levels (Langie et al. 2007) or even a direct effect of pH on DNA damage incision activity. Therefore, the conclusions drawn in this study need further mechanistic investigations. Secondly, some studies pointed out that the metabolic pathways may differ between normal cells and cancerous cells (Amoedo et al. 2013; Hockenbery et al. 2013). Under certain conditions of stress (including hypoxia or low pH), tumors may change their gene expression and adapt, whereas most normal cells may undergo apoptosis (Brooks et al. 2005; Hill et al. 2001; Park et al. 1999). Therefore, in current study, we included both a normal cell line (BEAS-2B cells) and a carcinoma cell line (A549) and similar effects of low pH combined with B[a]P exposure on CYP1A1 mRNA expression, EROD activity and B[a]P metabolism were observed, although at a different pH. BEAS-2B had a delay in their B[a]P metabolism already at pH 7.0, whereas the same effect was only observed in A549 cells at its most extreme pH condition (pH 5.5) after 48 h of incubation. Therefore, the delay in B[a]P metabolism by low pH seems stronger in normal cells than in cancer cells.

Our present results indicated that exposure of A549 and BEAS-2B cells to B[a]P at acidic pH results in lower *CYP1A1/CYP1B1* expression, EROD activity and DNA repair. This leads to inefficient B[a]P metabolism and subsequent accumulation of active B[a]P metabolites, B[a]P-related DNA adducts and overall DNA damage as assessed by B[a]P-related DNA adducts and γH2AX. At later time points, the pH_i_ and pH_e_ gradually restored toward pH 7.8, which increased enzyme activities and the cells started to metabolize B[a]P. Meanwhile, the unmetabolized B[a]P continuously triggers *CYP1A1* expression/activity possibly via an interaction with AhR. Moreover, DNA incision activity was not completely recovered in acidic samples after 24 h. In conclusion, our data demonstrate that acidic pH delayed metabolism of B[a]P and DNA repair activity. Consequently, higher DNA damage and DNA adduct formation were observed. Although we did not include all the enzymes which are involved in the metabolism of B[a]P, we showed a general molecular mechanism of how acidic pH influences the cellular response to the carcinogen B[a]P.

## Electronic supplementary material

Below is the link to the electronic supplementary material.

Supplementary material 1 (DOCX 29 kb)
