# Supplementary material for: Acidic cellular microenvironment modifies carcinogen-induced DNA damage and repair
Source: Arch Toxicol. 2016 Dec 22;91(6):2425–41. doi: 10.1007/s00204-016-1907-4 (PMC5429366; doi:10.1007/s00204-016-1907-4)
Supplement: Supplementary file 1 — Supplementary material 1 (DOCX 29 kb) [file 204_2016_1907_MOESM1_ESM.docx]

Supplemental Table 1. Concentration (nM) of B[a]P-9,10-diol, B[a]P-7,8-diol, B[a]P-3-OH and B[a]P at 6, 24 and 48 hours. Statistical comparison was performed between each pH condition (pH 7, 6.5, 6 and 5.5) with pH 7.8 at each time point.

| Cell  type | Metabolites or unmetabolised B[a]P | Time (h) | pH 7.8 | pH7 | pH 6.5 | pH 6 | pH 5.5 |
| --- | --- | --- | --- | --- | --- | --- | --- |
| A549 | B[a]P-9,10-diol | 6 | 155±4.3 | 87±12^***^ | 96±3.9^***^ | 26±0.7^***^ | 2.6±1.3^***^ |
|  |  | 24 | 1.3±0.6 | 3.9±1.5 | 5.2±0.2 | 77±6.8^***^ | 332±25.3^***^ |
|  |  | 48 | 0.8±0.1 | 0.6±0.2 | 2.9±0.9 | 2.8±0.4 | 269±2.5^***^ |
|  | B[a]P-7,8-diol | 6 | 30±1.2 | 19.7±2.0^***^ | 20.6±0.5^**^ | 9.3±0.9^***^ | 0.8±0.4^***^ |
|  |  | 24 | 0.3±0.1 | 0.4±0.2 | 1.5±0.02 | 4.0±0.3 | 86.6±6.2^***^ |
|  |  | 48 | 0.1±0.01 | 0.1±0.04 | 0.6±0.2 | 1.4±0.1 | 101±8.3^***^ |
|  | B[a]P-3-OH | 6 | 18.3±4.1 | 19.3±3.3 | 1.2±0.4^***^ | 0.9±0.2^***^ | 1.3±0.4^***^ |
|  |  | 24 | 1.3±0.2 | 0.8±0.2 | 1.7±0.3 | 4.5±0.9 | 1.5±0.1 |
|  |  | 48 | 0.9±0.1 | 0.6±0.03 | 3.2±0.07 | 2.5±0.02 | 8.5±0.2^***^ |
|  | B[a]P | 6 | 787±47 | 922±88 | 688±31 | 661±65 | 905±141 |
|  |  | 24 | 10.2±4.6 | 22±2.4 | 57.6±13 | 177±24^***^ | 509±20^***^ |
|  |  | 48 | 5.2±0.1 | 14.5±1.8 | 37.7±7.1^*^ | 143±6.5^***^ | 383±14^***^ |
| BEAS-2B | B[a]P-9,10-diol | 6 | 66.2±4.3 | 52.5±2.7 | 30.3±2.9^***^ |  |  |
|  |  | 24 | 109±6 | 142±12 | 207±6^***^ |  |  |
|  |  | 48 | 70±7 | 131.7±15.3^**^ | 220±4^***^ |  |  |
|  | B[a]P-7,8-diol | 6 | 45.5±2.7 | 37.1±0.4^*^ | 27±1.7^***^ |  |  |
|  |  | 24 | 13.3±0.4 | 62.4±2^***^ | 76.4±4.2^***^ |  |  |
|  |  | 48 | 2.3±0.2 | 22.7±0.1^***^ | 111±1.7^***^ |  |  |
|  | B[a]P-3-OH | 6 | 22.5±2.1 | 32.4±1.7^**^ | 7.8±0.8^***^ |  |  |
|  |  | 24 | 19.3±0.9 | 24.6±1.5^*^ | 28.2±1.2^**^ |  |  |
|  |  | 48 | 16.1±0.6 | 17.9±2.6 | 45.3±5.8^***^ |  |  |
|  | B[a]P | 6 | 500±22 | 663±10^***^ | 823±18^***^ |  |  |
|  |  | 24 | 33.2±0.5 | 52.4±2 | 458±19^***^ |  |  |
|  |  | 48 | 7.8±0.5 | 24±2.5 | 209±12^***^ |  |  |

^*^p<0.05

^**^p<0.01

^***^p<0.001

Supplemental Table 2 Raw data of DNA incision activity based on tail moment (TM) and tail intensity (TI) for both A549 and BEAS-2B cell lines. Median of the raw data were extracted and incision activity was calculated according to the formula in Langie et al. (2006). Data shown are the mean ± SEM of 4 independent samples (n=4).

| Cell | TM or TI | Time (h) | 7.8 | 7.0 | 6.5 | 6.0 | 5.5 |
| --- | --- | --- | --- | --- | --- | --- | --- |
| A549 | Tail Moment | 6 | 2.06±0.2 | 1.80±0.05 | 0.47±0.37 | 0.23±0.02 | 0.39±0.25 |
|  |  | 24 | 3.39±0.93 | 1.58±0.06 | 1.74±0.19 | 0.31±0.14 | 1.01±0.49 |
|  |  | 48 | 0.96±0.09 | 1.18±0.22 | 1.11±0.16 | 1.30±0.10 | 1.05±0.11 |
|  | Tail Intensity | 6 | 6.62±1.48 | 5.37±1.32 | 3.06±0.49 | 3.17±2.30 | 2.87±1.06 |
|  |  | 24 | 9.36±0.25 | 5.88±1.48 | 5.59±1.78 | 3.50±0.11 | 4.19±0.18 |
|  |  | 48 | 3.23±0.41 | 4.56±0.20 | 4.73±0.30 | 5.90±0.29 | 7.84±0.63 |
| BEAS-2B | Tail Moment | 6 | 2.47±1.05 | 1.34±0.25 | 0.71±0.19 |  |  |
|  |  | 24 | 3.78±1.47 | 1.95±0.24 | 1.37±1.17 |  |  |
|  |  | 48 | 1.88±0.24 | 3.07±0.81 | 5.22±1.44 |  |  |
|  | Tail Intensity | 6 | 15.39±6.52 | 12.45±3.61 | 5.84±3.23 |  |  |
|  |  | 24 | 21.13±3.93 | 15.45±1.50 | 8.29±5.60 |  |  |
|  |  | 48 | 10.79±1.68 | 15.16±7.77 | 23.10±3.43 |  |  |

Supplemental Table 3. Nonparametric Spearmen’s rho correlation analysis of different variables (B[a]P-7,8-diol, CYP1A1 mRNA expression, CYP1B1 mRNA expression, EROD activity, DNA incision activity and DNA adducts) derived from experiments with A549 cells.

| Cell type |  |  | B[a]P-7,8-diol | CYP1A1 mRNA expression | CYP1B1 mRNA expression | EROD activity | DNA incision activity | DNA adducts |
| --- | --- | --- | --- | --- | --- | --- | --- | --- |
| A549 | B[a]P-7,8-diol | Correlation Coefficient |  | .704^**^ | .914^***^ | .607^*^ | -.121 | .555^*^ |
|  |  | Sig. (2-tailed) |  | .003 | .0001 | .016 | .666 | .032 |
|  | CYP1A1 mRNA expression | Correlation Coefficient | .704^**^ |  | .807^***^ | .679^**^ | .0001 | -.400 |
|  |  | Sig. (2-tailed) | .003 |  | .0001 | .005 | 1.000 | .140 |
|  | CYP1B1 mRNA expression | Correlation Coefficient | .914^***^ | .807^***^ |  | .514^*^ | -.154 | -.239 |
|  |  | Sig. (2-tailed) | .0001 | .0001 |  | .050 | .585 | .390 |
|  | EROD activity | Correlation Coefficient | .607^*^ | .679^**^ | .514^*^ |  | .371 | .289 |
|  |  | Sig. (2-tailed) | .016 | .005 | .050 |  | .173 | .296 |
|  | DNA incision activity | Correlation Coefficient | -.121 | .0001 | -.154 | .371 |  | .164 |
|  |  | Sig. (2-tailed) | .666 | 1.000 | .585 | .173 |  | .558 |
|  | DNA adducts | Correlation Coefficient | . 555^*^ | -.400 | -.239 | .289 | .164 |  |
|  |  | Sig. (2-tailed) | .032 | .140 | .390 | .296 | .558 |  |

***. Correlation is significant at the 0.001 level (2-tailed).

**. Correlation is significant at the 0.01 level (2-tailed).

*. Correlation is significant at the 0.05 level (2-tailed).

Supplemental Table 4. Nonparametric Spearmen’s rho correlation analysis of different variables (B[a]P-7,8-diol, CYP1A1 mRNA expression, CYP1B1 mRNA expression, EROD activity, DNA incision activity and DNA adducts) derived from experiments with BEAS-2B cells.

| Cell type |  |  | B[a]P-7,8-diol | CYP1A1 mRNA expression | CYP1B1 mRNA expression | EROD activity | DNA incision activity | DNA adducts |
| --- | --- | --- | --- | --- | --- | --- | --- | --- |
| BEAS-2B | B[a]P-7,8-diol | Correlation Coefficient |  | .750^*^ | .800^**^ | .733^*^ | .100 | .717^*^ |
|  |  | Sig. (2-tailed) |  | .020 | .010 | .025 | .798 | .030 |
|  | CYP1A1 mRNA expression | Correlation Coefficient | .750^*^ |  | .517 | .883^**^ | -.067 | -.167 |
|  |  | Sig. (2-tailed) | .020 |  | .154 | .002 | .865 | .668 |
|  | CYP1B1 mRNA expression | Correlation Coefficient | .800^**^ | .517 |  | .633 | -.167 | .067 |
|  |  | Sig. (2-tailed) | .010 | .154 |  | .067 | .688 | .865 |
|  | EROD activity | Correlation Coefficient | .733^*^ | .883^**^ | .633^*^ |  | .033 | -.117 |
|  |  | Sig. (2-tailed) | .025 | .002 | .067 |  | .932 | .765 |
|  | DNA incision activity | Correlation Coefficient | .100 | -.067 | -.167 | .033 |  | .317 |
|  |  | Sig. (2-tailed) | .798 | .865 | .668 | .932 |  | .406 |
|  | DNA adducts | Correlation Coefficient | . 717^*^ | -.167 | .067 | -.117 | .317 |  |
|  |  | Sig. (2-tailed) | .030 | .668 | .865 | .765 | .406 |  |

***. Correlation is significant at the 0.001 level (2-tailed).

**. Correlation is significant at the 0.01 level (2-tailed).

*. Correlation is significant at the 0.05 level (2-tailed).
